# Advances in the Understanding of Frontotemporal Dementia

**DOI:** 10.3390/cells12050781

**Published:** 2023-03-01

**Authors:** Rina Bandopadhyay, Ariana Gatt, Tammaryn Lashley

**Affiliations:** 1 Reta Lila Weston Institute of Neurological Studies, Department of Clinical and Movement Neuroscience, UCL Institute of Neurology, 1 Wakefield Street, London WC1N 1PJ, UK; 2 Queen Square Brain Bank, Department of Neurodegenerative Diseases, UCL Institute of Neurology, 1 Wakefield Street, London WC1N 1PJ, UK

Frontotemporal dementia (FTD) encompasses a group of clinically, genetically and pathologically heterogeneous neurodegenerative disorders that mainly affect people under the age of 64 years. However, around 25% of those affected have a later age of onset. FTD represents 10–20% of all dementia cases [1]. It is predominantly characterized by the progressive atrophy of the frontal and temporal lobes [2]. Disease duration ranges between 2 and 20 years, with 8 years being the mean following the onset of symptoms. FTD treatment is restricted to symptom control, and no disease-modifying treatments are available.

The clinical hallmarks of FTD include gradual yet progressive deficits in behaviour and/or language with the relative preservation of memory. Subtypes of FTD are identified clinically according to the symptoms that appear prominently at presentation. Clinical diagnoses include behavioral variant FTD (bVFTD), which accounts for nearly 60% of cases; primary progressive aphasia (PPA), which affects language; and the movement disorders progressive supranuclear palsy (PSP) and corticobasal syndrome (CBS) [2]. With disease progression, the debilitating symptoms cause marked impairments of social and/or occupational functioning. Early and accurate diagnosis is crucial for the streamlining and development of any disease-modifying treatment therapies.

A third of FTD cases are genetically linked with mutations occurring in *C9orf72*, progranulin (*GRN*) and *MAPT*, with *C9orf72* repeat expansions being the most common. Neuropathologically, TAR DNA binding protein-43 (TDP-43), fused in sarcoma (FUS) and tau are three major proteins that cause pathological deposits in FTD post-mortem brains [2]. Those with *C9orf72* expansions also have an additional pathology where di-peptide repeat proteins are also found deposited in patients. It is thought that disease pathogenesis is caused either by a gain of toxic function or a loss of nuclear function associated with protein dislocation from the nucleus, which in turn may lead to neuronal degeneration. FUS regulates the transcription of multiple genes, including the *MAPT* gene [3,4]. Recent studies have highlighted the molecular pathways associated with lysosomal dysfunction, lipid dysregulation, RNA splicing aberrations, synaptic loss and neuroinflammation as putative causes of sporadic forms of FTD. The disease mechanisms are far from understood in FTD, especially the cellular changes occurring in the early disease stages. Continued research with improved animal models, iPS technologies, clinicopathological correlations with donated human brain tissue and the discovery of early biomarkers of disease progression should enable us to rationalize the mechanisms involved in these neurodegenerative diseases and identify much-needed therapeutic targets. This Special Issue will collect articles relating to all advances in FTD research, both clinical and non-clinical. 
